# An Adaptive-Output Operational Amplifier for Electrostatic Closed-Loop MEMS Gyroscope Drive Circuits

**DOI:** 10.3390/mi17080900

**Published:** 2026-07-27

**Authors:** Xiaoqin Li, Wanting Rong, Diqun Yan, Xiali Han, Shanshan Wang, Wenbo Zhang, Hao Ye, Xiangyu Li

**Affiliations:** 1 School of Artificial Intelligence, Ningbo Polytechnic University, Ningbo 315800, China; xqli@nbpu.edu.cn; 2 School of Intelligent Manufacturing, Huzhou College, Huzhou 313000, China; rongwanting1@163.com; 3 College of Artificial Intelligence, Ningbo University of Finance and Economics, Ningbo 315175, China; 4 Institute of Drug Discovery Technology, Ningbo University, Ningbo 315211, China; 5 School of Astronautics, Harbin Institute of Technology, Harbin 150001, China; 6 College of Electrical and Electronic Engineering, Wenzhou University, Wenzhou 325035, China; yehao@wzu.edu.cn; 7 Ningbo Institute of Digital Twin, Eastern Institute of Technology, Ningbo 315042, China; 8 Ningbo Key Laboratory of Spatial Intelligence and Digital Derivative, Ningbo 315200, China

**Keywords:** front-end circuits, MEMS gyroscope, continuous-time voltage detection circuit

## Abstract

To address the challenge that microelectromechanical system (MEMS) gyroscope electrostatic force-modulated closed-loop self-excited driving circuits experience significant dynamic variations in capacitive load and driving demand under different operating conditions, such as start-up, steady-state resonance maintenance, and environmental perturbations, making it difficult to simultaneously achieve strong driving capability, stable oscillation, and low power consumption, this paper proposes a high-energy-efficiency adaptive output operational amplifier architecture. Based on a dynamic load-sensing mechanism, the design introduces a three-threshold decision scheme combining a high threshold, a low threshold, and a mid-supply reference voltage. By coordinating a continuous-time voltage detection circuit with a bidirectional shift register, the proposed approach enables accurate identification of the output state and the load level. A time-division-multiplexed two-stage control strategy is adopted to rapidly compensate for the drive capability under abrupt load changes, while proactively disabling redundant output units under steady-state conditions, thereby achieving power delivery on demand. The output stage employs a Class-AB push–pull structure integrating an improved low-leakage single-pole double-throw (SPDT) switch, which hard shuts off the power transistors in the non-operating state to effectively eliminate the subthreshold leakage current. Circuit simulations in a 0.18 μm CMOS process demonstrate that the proposed operational amplifier can adaptively regulate its output current in real time according to variations in the gyroscope driving demand, ensuring sufficient an electrostatic driving force and oscillation stability during transient conditions while significantly reducing static power consumption during the resonance steady state. The proposed design provides an effective solution for high-performance and high-energy-efficiency interface circuit design in MEMS gyroscope electrostatic force-modulated closed-loop self-excited driving systems.

## 1. Introduction

With the rapid development of advanced intelligent manufacturing and precision engineering, high-performance sensing and control technologies have become increasingly important for achieving accurate motion control and system reliability [1,2,3,4,5,6,7,8]. It has become important tools for solving complex scientific and engineering problems, providing new opportunities for the design and optimization of integrated circuits and MEMS systems [9,10,11,12]. Integrated circuits play a particularly important role in sensor applications, especially in high-performance MEMS interface systems [13,14,15]. In practical applications, electrostatic force-modulated closed-loop self-excited driving circuits for MEMS gyroscopes operate under highly dynamic driving conditions. During start-up and resonance establishment, the drive loop must provide strong transient electrostatic driving capability to rapidly excite the proof mass into stable oscillation. Under steady-state resonance conditions, however, the drive demand decreases significantly, and excessive power consumption should be avoided to improve energy efficiency and thermal stability. Furthermore, variations in the environmental temperature, mechanical disturbances, quality factor (Q-factor), and structural parameter drift may continuously alter the resonant characteristics of the gyroscope, thereby imposing stringent requirements on the driving capability and power adaptability of the interface circuit.

In particular, in high-precision inertial sensing systems, precision signal acquisition, sensor interface circuits, and closed-loop resonant control systems [16], the performance of the front-end operational amplifier in the electrostatic drive loop is especially critical. However, existing operational amplifier designs often struggle to simultaneously achieve strong transient drive capability, stable resonance maintenance, and low power consumption [17,18,19,20,21,22,23,24,25,26]. Conventional static output-stage architectures are typically designed for worst-case transient driving conditions to guarantee start-up reliability and disturbance rejection, which inevitably leads to considerable power redundancy during steady-state oscillation. Although several techniques, such as slew-rate-enhancement structures, adaptive biasing, or programmable-gain amplification, improve the transient response or gain flexibility to some extent, their output stages generally remain fixed and cannot dynamically regulate the number of enabled power devices according to real-time variations in the gyroscope drive demand [27]. This fixed-drive strategy not only degrades energy efficiency, but may also result in an insufficient electrostatic driving force or delayed response under abrupt perturbations and resonance fluctuations. The main contribution of this work is an adaptive-output operational amplifier specifically designed for the electrostatic closed-loop drive path of an MEMS gyroscope. The novelty is not the use of a standalone comparator, shift register, or output switch, but the drive-loop-oriented integration of these blocks into an autonomous output-strength regulation mechanism. Different from adaptive-bias amplifiers, the proposed circuit does not primarily regulate the bias current of the core gain stage. Different from slew-rate-enhancement amplifiers, the adaptive action is not limited to transient current injection for improving the large-signal slew rate. Different from conventional programmable output-stage amplifiers, the output-stage control word is not externally assigned as a static configuration. Instead, the proposed three-threshold voltage-detection circuit monitors the output-voltage state with respect to RefH, RefM, and RefL, and the bidirectional shift register retains and updates the enabled output-array state in finite steps. The adaptive Class-AB output array therefore increases the available drive strength under under-drive conditions and disables redundant output units after load relaxation. In addition, the improved SPDT gate-clamping switch forces the gates of disabled PMOS and NMOS power devices to VDD and GND, respectively, thereby suppressing unnecessary static conduction in idle output units. This work is therefore positioned as a drive-loop operational-amplifier block for electrostatic closed-loop MEMS gyroscopes rather than as a complete MEMS gyroscope readout system. Although adaptive-bias amplifiers and slew-rate enhancement techniques have been extensively investigated to improve transient performance and power efficiency, these approaches generally adjust the bias current while maintaining a fixed output-stage structure. In contrast, the proposed architecture dynamically reconfigures segmented output units according to the required driving capability, enabling adaptive power reduction during steady-state operation while preserving sufficient output current during start-up and transient conditions.

## 2. Proposed Structure

**Figure 1:**
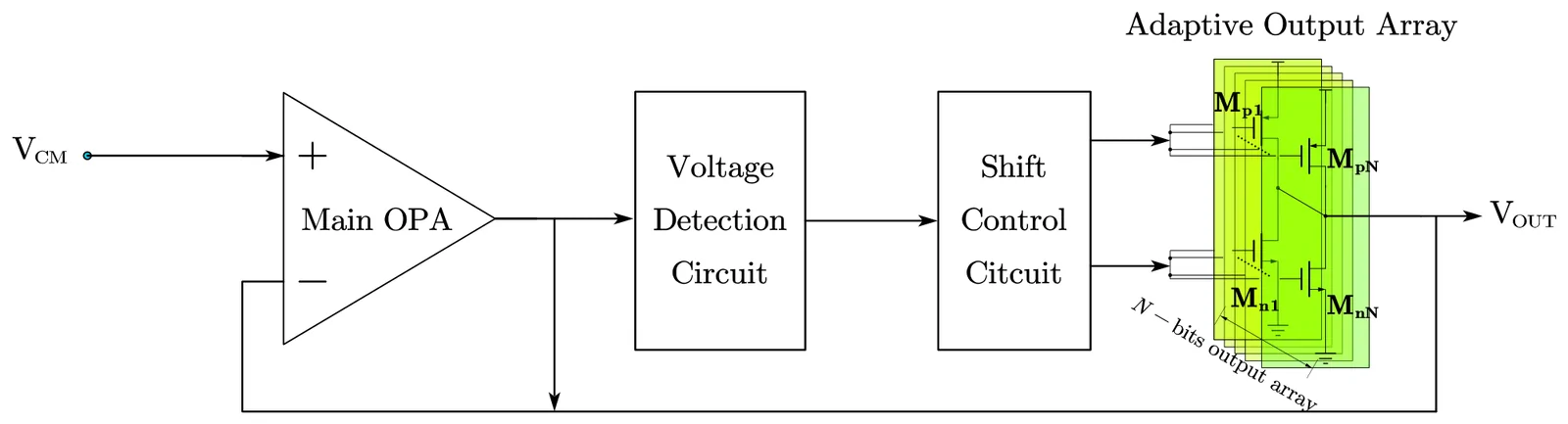
Proposed adaptive output operational amplifier structure.

The proposed adaptive-output operational amplifier architecture is shown in Figure 1. The structure is straightforward and mainly consists of a main operational amplifier, a voltage-detection circuit, a shift-control circuit, and an adaptive-output array, where the main amplifier and the output array form a negative-feedback loop. When the external load requires the operational amplifier to deliver current, the voltage-detection circuit continuously samples the output-voltage variation at the amplifier output and determines the current operating state through internal continuous-time comparators. It controls the bidirectional shift register in the shift-control circuit to switch the output strategy. By receiving the “0” or “1” signal generated by the voltage-detection circuit, the bidirectional shift register dynamically adjusts the number of enabled PMOS/NMOS units in the output array. The shift register outputs a multi-bit digital control word, where each bit controls the switching of one output unit transistor. In this way, different numbers of unit transistors can be turned on in parallel, thereby adjusting the output-driving capability.

The proposed regulation scheme should be distinguished from conventional output-current enhancement techniques. In this work, the output stage is treated as a digitally retained, drive-strength-scalable array rather than a fixed-size Class-AB stage assisted by temporary bias boosting. The voltage-detection circuit evaluates the output-voltage deviation relative to three reference levels and converts the analog drive-demand information into discrete control decisions. The bidirectional shift-control circuit then updates the number of enabled output units only when the output voltage leaves the predefined threshold window. Once the output voltage returns to the RefL–RefH window, the current enable state is retained. This finite-step and state-retention behavior is essential for avoiding unnecessary output-stage switching during steady-state resonant operation.

Since conventional detection circuits can only passively increase the drive strength when the output voltage drops under heavy load, yet cannot proactively sense load relaxation and reduce redundant drive current, this design introduces three key reference voltages: the high threshold *Ref_H_*, the low threshold *Ref_L_*, and the mid-supply reference voltage *Ref_M_*. The voltage detection circuit comprises three continuous-time comparators followed by combinational logic. Based on the relationship between *V_out_* and these thresholds, the system classifies its operating condition into three regions. The operating states of the proposed system are classified into three distinct regions according to the relationship between the output voltage and the predefined reference thresholds. In the stable region, where *Ref_L_* < *V_out_* < *Ref_H_*, the available drive capability is sufficient to maintain loop stability, and no significant adjustment of the output stage is required. When *V_out_* falls below *Ref_L_*, the system enters the under-drive region, indicating that the output stage is unable to satisfy the current load demand; in this case, additional transient current must be supplied to restore the output voltage. Furthermore, a load-decision region is defined by introducing a common-mode reference threshold *Ref_M_*. During the subsequent detection period, the comparison between *V_out_* and *Ref_M_* enables the system to differentiate between a sustained heavy-load condition and a light-load over-drive condition, thereby providing a quantitative basis for adaptive power regulation and energy-efficiency optimization.

To ensure system stability, this paper proposes a time-division-multiplexed two-stage regulation mechanism. The total number of adaptive array units is defined as *N*, satisfying *N* = 2*^j^* (*j* > 4). To balance the response speed and stability, the detection period *T_det_* and the shift-control period *T_ctrl_* are set to satisfy the following timing constraint:

(1) $$\left\{\begin{array}{l} K=\frac{N}{2}-1\\ T_{det}=K\times T_{ctrl} \end{array}\right.$$

Here, *K* denotes the maximum number of adjustment steps allowed within a single detection window. This design ensures that, under abrupt load variations, the circuit has a sufficient time window to perform progressive adjustments, thereby avoiding the output oscillations caused by overly aggressive responses. In each operating cycle, the control logic executes in two phases according to the timing sequence. Let Δ*M*[*n*] denote the change in the number of activated units during the n-th control period.

In Phase 1, i.e., during the first detection period, the system primarily checks whether the output voltage deviates excessively, with the goal of quickly pulling the output voltage back to the target range, as follows:

(2) $$\Delta M\left[n\right]=\left\{\begin{array}{ll} +1, & V_{out}<Ref_{L}\left(\mathrm{Pull}\;\mathrm{Up}\right)\\ +1, & V_{out}>Ref_{H}\left(\mathrm{Pull}\;\mathrm{Down}\right)\\ 0, & Ref_{L}\leq V_{out}\leq Ref_{H} \end{array}\right.$$

In this phase, once an out-of-range output voltage is detected, the shift register performs finite stepwise shifting at the control-clock edges for up to K cycles, enabling the output units one by one until the output voltage returns to the safe RefL–RefH window.

In Phase 2, i.e., during the second detection period, the system compares the output with *Ref_M_* to make a second decision, leading to the following:

(3) $$\Delta M\left[n\right]=\left\{\begin{array}{ll} +1, & V_{out}\leq Ref_{M}\left(\mathrm{High}\;\mathrm{Load}\right)\\ -1, & V_{out}>Ref_{M}\left(\mathrm{Low}\;\mathrm{Load}/\mathrm{Over}\;\mathrm{Drive}\right)\\ 0, & \mathrm{Other} \end{array}\right.$$

This logic indicates that if *V_out_* remains higher than *Ref_M_* in the pull-current mode, the available drive capability is excessive relative to the load. In this case, the system performs a reverse shifting operation to progressively turn off redundant power units, thereby achieving power delivery on demand.

**Figure 2:**
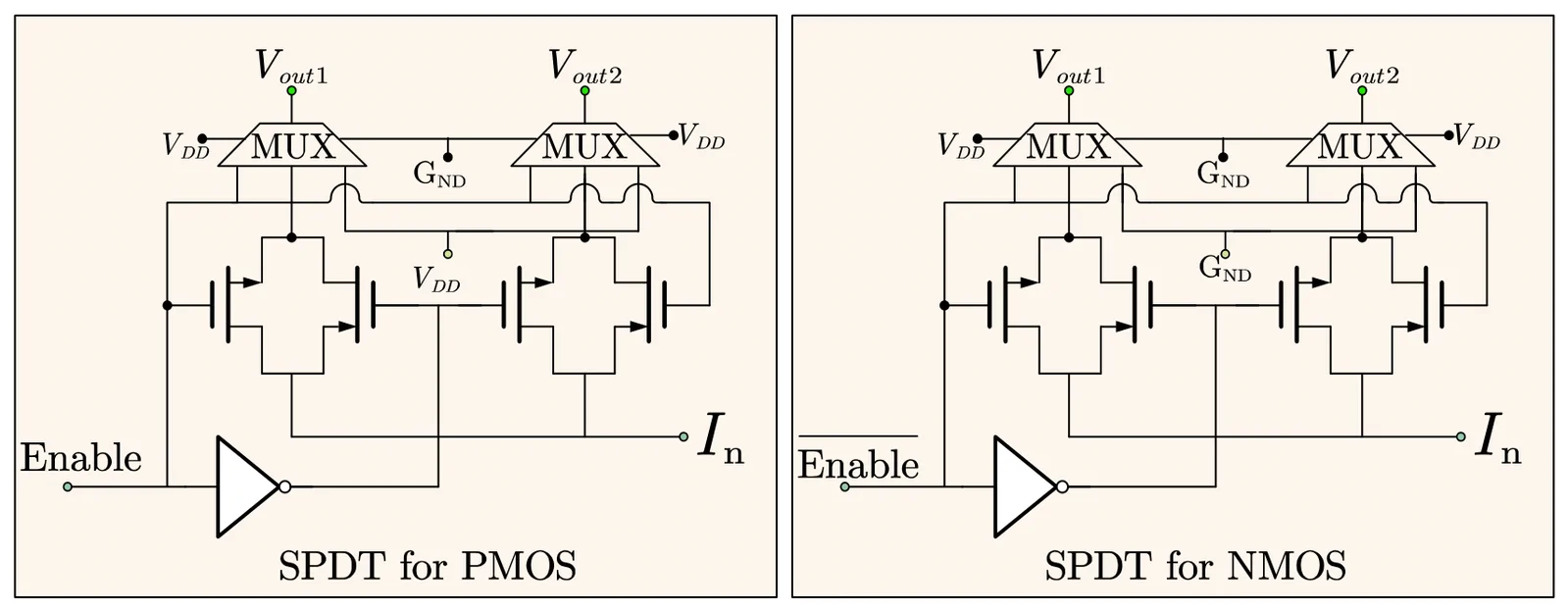
Improved SPDT switch (single-pole double-throw).

The output array adopts a Class-AB push–pull output stage, which offers low power consumption and strong drive capability. Here, M_p_/M_n_ form the static output stage, while P_out_/N_out_ constitute the adaptive output stage. The drive strength can be controlled by adjusting the number of transistor fingers, and the total output current is jointly provided by the static output stage and *N* parallel adaptive units, as expressed by the following:

(4) $$I_{Sum\text{\_}out}(t)=I_{n}+\sum_{i=1}^{M\left(t\right)}I_{out}$$

where $I_{n}$ denotes the current of a single output unit, and $I_{out}$ represents the total output current formed by the parallel combination of multiple units. To further reduce standby power consumption, each output unit integrates an improved single-pole double-throw (SPDT) switch module. Unlike conventional approaches that merely disconnect the signal path, this design implements a hard shut-off function using a multiplexer and an inverter. When an output unit is determined to be in the “static” state, the SPDT switch forcibly pulls the gate of the PMOS power transistor to V_DD_ and the gate of the NMOS power transistor to GND. This completely eliminates leakage current of the power devices in the subthreshold region, ensuring that the static power of idle array units under light-load operation approaches zero, thereby significantly improving the overall energy efficiency. The improved SPDT switch is shown in Figure 2.

## 3. Design and Implementation of Key Circuit Modules

**Figure 3:**
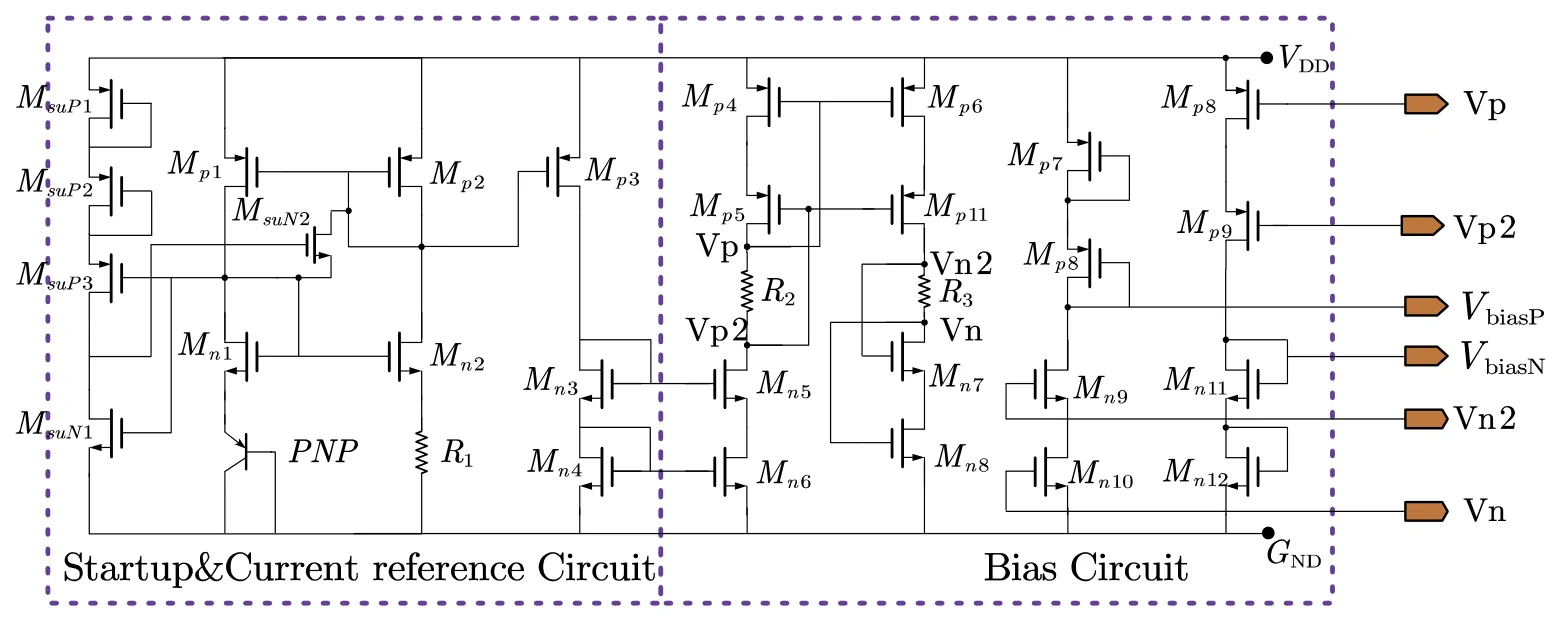
Schematic of bias circuit.

**Figure 4:**
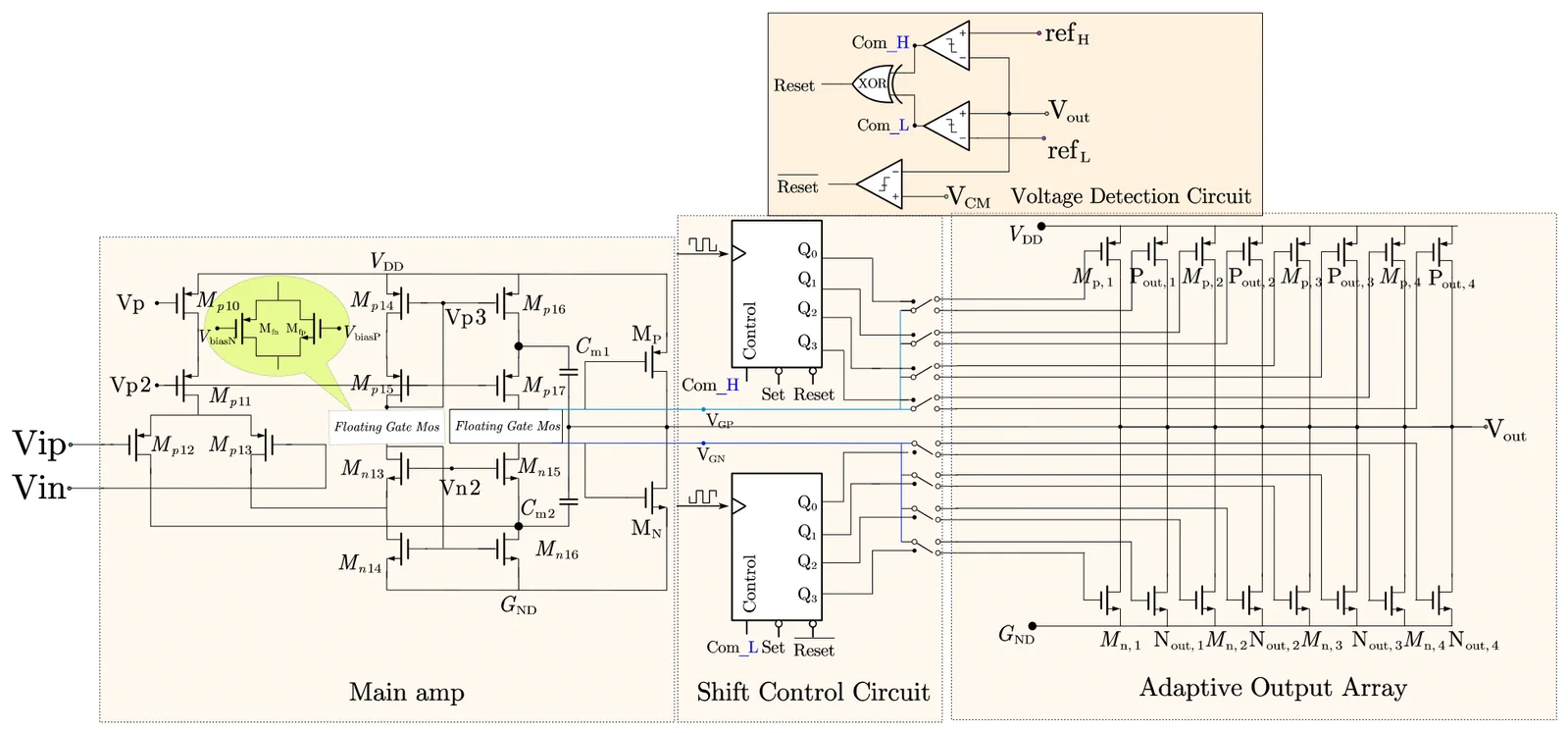
Schematic of the adaptive-output operational amplifier.

This work presents the transistor-level implementation of the key modules, including the main amplifier stage, a high-speed low-power continuous-time comparator, and an output array with low-leakage switches. The detailed circuit schematic is shown in Figure 3 and Figure 4. In this design, a commonly used PMOS-input folded-cascode operational amplifier architecture is employed. This module mainly consists of a startup circuit, a current reference circuit, a bias circuit, and a folded-cascode amplifier. Based on small-signal analysis, the gain of this structure can be expressed as follows:

(5) $$\begin{array}{c} A_{v,total}=A_{v1}\cdot A_{v2}\\ A_{v1}\approx -g_{mp12}\left[g_{mn14}r_{on14}\left(r_{op12}∥r_{on13}\right)//\left(g_{mp14}r_{op14}r_{op13}\right)\right]\\ A_{v2}\approx -m\left(g_{mp_{out}}+g_{mn_{out}}\right)\frac{r_{oPout}//r_{oNout}}{m} \end{array}$$

Here, *m* represents the number of transistors turned on in the array, *gm_p_*_12_ denotes the transconductance of the input differential pair, and *r_o_* is the output resistance. This stage provides the dominant low-frequency gain, ensuring the accuracy of the closed-loop system, and drives the subsequent voltage detection circuit.

To accurately capture small voltage deviations within a nanosecond time scale and effectively suppress kickback noise, an improved three-stage cascaded current-comparator architecture is adopted. This topology mainly consists of an input preamplifier stage, a positive-feedback decision latch stage, and an output buffer stage. The preamplifier as shown in Figure 4 is formed by transistors M_1_~M_7_. Transistors M_5_ and M_6_ sense the output voltage, which is converted into current through M_2_ and M_3_. By leveraging the replication property of the current mirror, the input voltage variation is translated into constant drive currents *I*_1_ and *I*_2_, which are injected into the subsequent positive-feedback decision latch stage.

**Figure 5:**
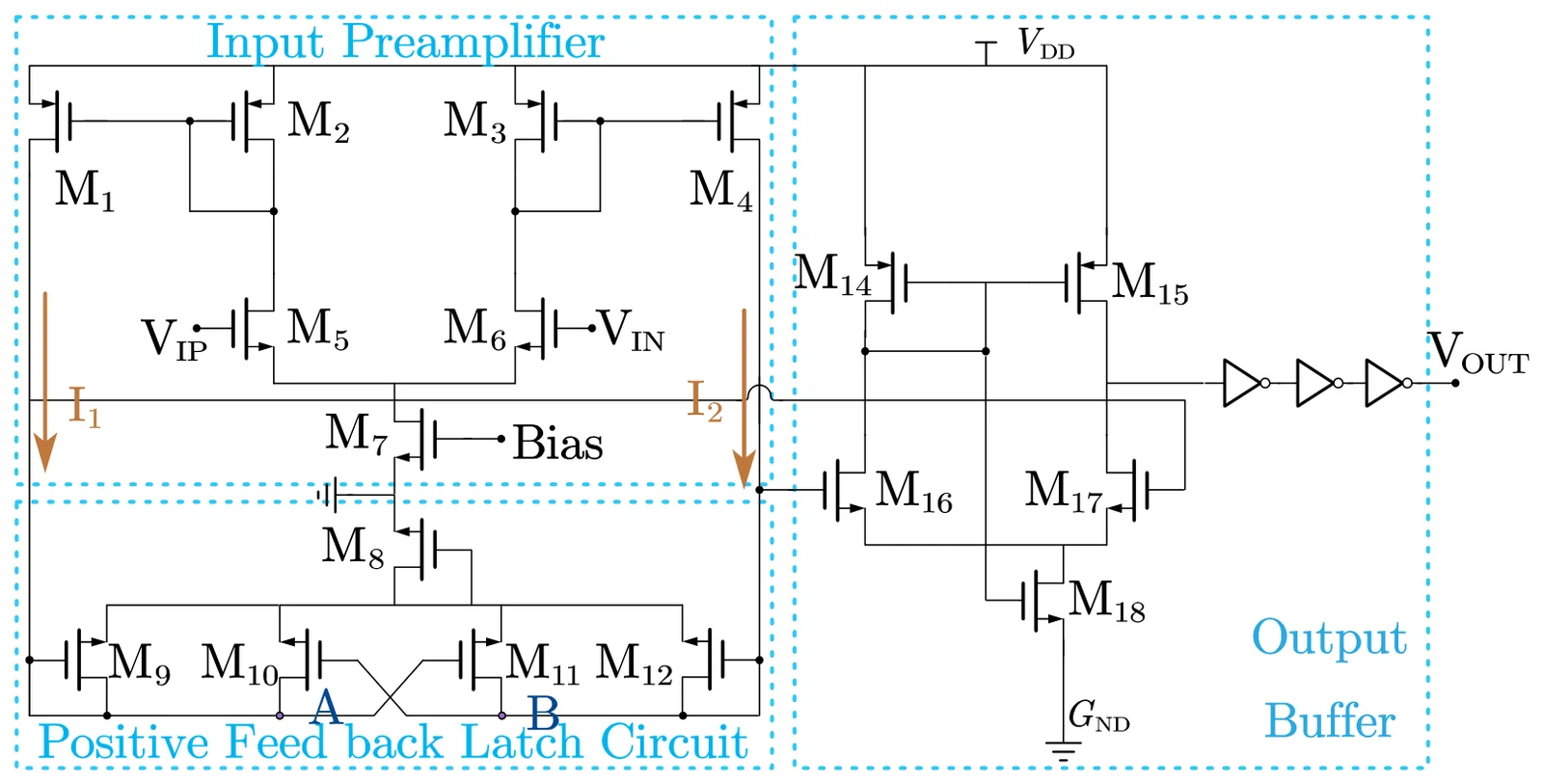
High-speed low-power continuous-time comparator.

The positive-feedback decision latch stage consists of a positive-feedback loop formed by transistors M8~M12 as shown in Figure 5. The circuit exploits the positive-feedback gain provided by the cross-coupled structure to accelerate the voltage transition at the internal nodes. The voltage response at latch nodes C and D depends on the degree of imbalance between the input currents. In the initial state, assuming that the circuit is perfectly symmetric, i.e., *g_m_*_9_ = *g_m_*_12_ and *g_m_*_10_ = *g_m_*_11_, Kirchhoff’s current law yields the small-signal behavior of the node voltages as follows:

(6) $$\left\{\begin{array}{l} v_{C}=\frac{g_{m9}}{g_{m9}^{2}-g_{m11}^{2}}\left[I_{1}-\frac{g_{m11}}{g_{m9}}I_{2}\right]\\ v_{D}=\frac{-g_{m11}}{g_{m9}^{2}-g_{m11}^{2}}\left[I_{1}-\frac{g_{m9}}{g_{m11}}I_{2}\right] \end{array}\right.$$

Here, g_m9_ and g_m10_ denote the transconductances of the load transistors and the cross-coupled transistors, respectively, while I1 and I2 are the injected currents converted from the input voltage. Similarly, the large-signal behavior of this structure can be expressed as follows:

(7) $$\left\{\begin{array}{l} V_{A}=V_{th}+\sqrt{\frac{K_{n9}}{K_{n9}^{2}-K_{n11}^{2}}\left(I_{1}-\frac{K_{n11}}{K_{n9}}I_{2}\right)}\\ V_{B}=V_{th}+\sqrt{\frac{K_{n11}}{K_{n9}^{2}-K_{n11}^{2}}\left(\frac{K_{n9}}{K_{n11}}I_{2}-I_{1}\right)} \end{array}\right.$$

where $K_{n}=\frac{1}{2}\mu C_{ox}\frac{W}{L}$. This expression indicates that the node voltages vary nonlinearly with the input current difference. When *g_m_*_9_ > *g_m_*_11_, the positive-feedback mechanism of this structure rapidly amplifies the branch current mismatch; this condition can be achieved by adjusting the *W/L* ratios of M_9_~M_12_. The output buffer stage consists of transistors M_14_~M_17_ and a three-stage inverter chain. The self-biased OTA formed by M_14_~M_17_ increases the comparator’s conversion gain and effectively suppresses common-mode noise, while the inverters mainly serve as a digital buffer to quantize the OTA’s analog output into digital logic levels for controlling the subsequent shift-register logic.

**Figure 6:**
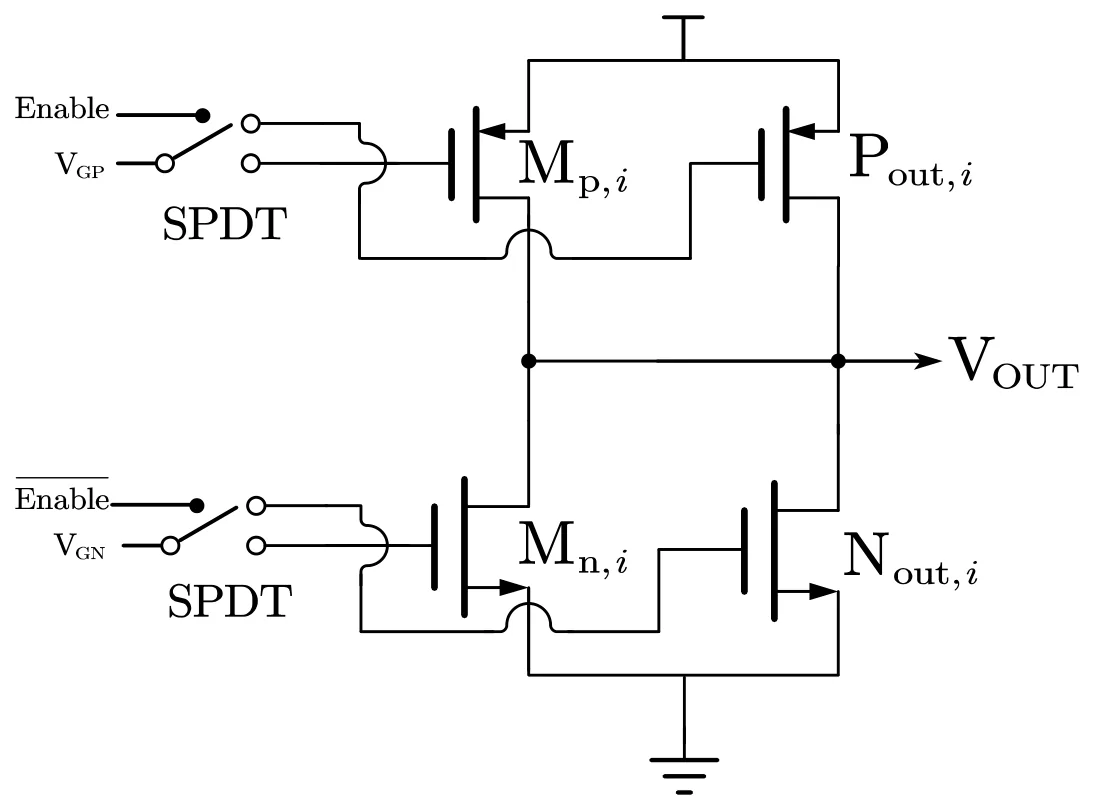
Adaptive output unit.

As shown in Figure 6, the adaptive output array consists of N adaptive-output units connected in parallel. Each adaptive unit contains a pair of static output transistors, M_p,i_ and M_n,i_, together with a pair of Class-AB push–pull output transistors, namely the pull-up PMOS power transistor P_out,i_ and the pull-down NMOS power transistor N_out,i_. The drains of these four transistors are directly connected to the output node Vout of the operational amplifier. The improved single-pole double-throw (SPDT) switch is placed in the gate-control path of the output-unit transistors and is used to select the connection state of the transistor gates. When the adaptive unit is enabled, the SPDT switch connects the gates of P_out,i_ and N_out,i_ to the PMOS and NMOS gate-drive signals generated by the main output stage, respectively, namely the floating gate voltages V_GP_ and V_GN_ produced by the push–pull stage of the preceding operational amplifier. As a result, the unit participates in Class-AB push–pull amplification. In this operating state, the output current is provided through the source–drain paths of P_out,i_ or N_out,i_, rather than being transmitted through the SPDT switch itself. Specifically, when the output node requires pull-up current, the enabled PMOS output unit supplies the current to Vout. Conversely, when the output node requires pull-down current, the enabled NMOS output unit sinks the current from Vout. Under light-load or standby conditions, the digital control signal generated by the shift register disables redundant adaptive units. In this case, the SPDT switch clamps the gate of the PMOS power transistor to VDD and the gate of the NMOS power transistor to GND, ensuring that both P_out,i_ and N_out,i_ are strongly turned off. Through this gate-clamping mechanism, the subthreshold leakage current of idle output units is effectively suppressed, while the introduction of additional series switch resistance at the main output node is avoided.

**Figure 7:**
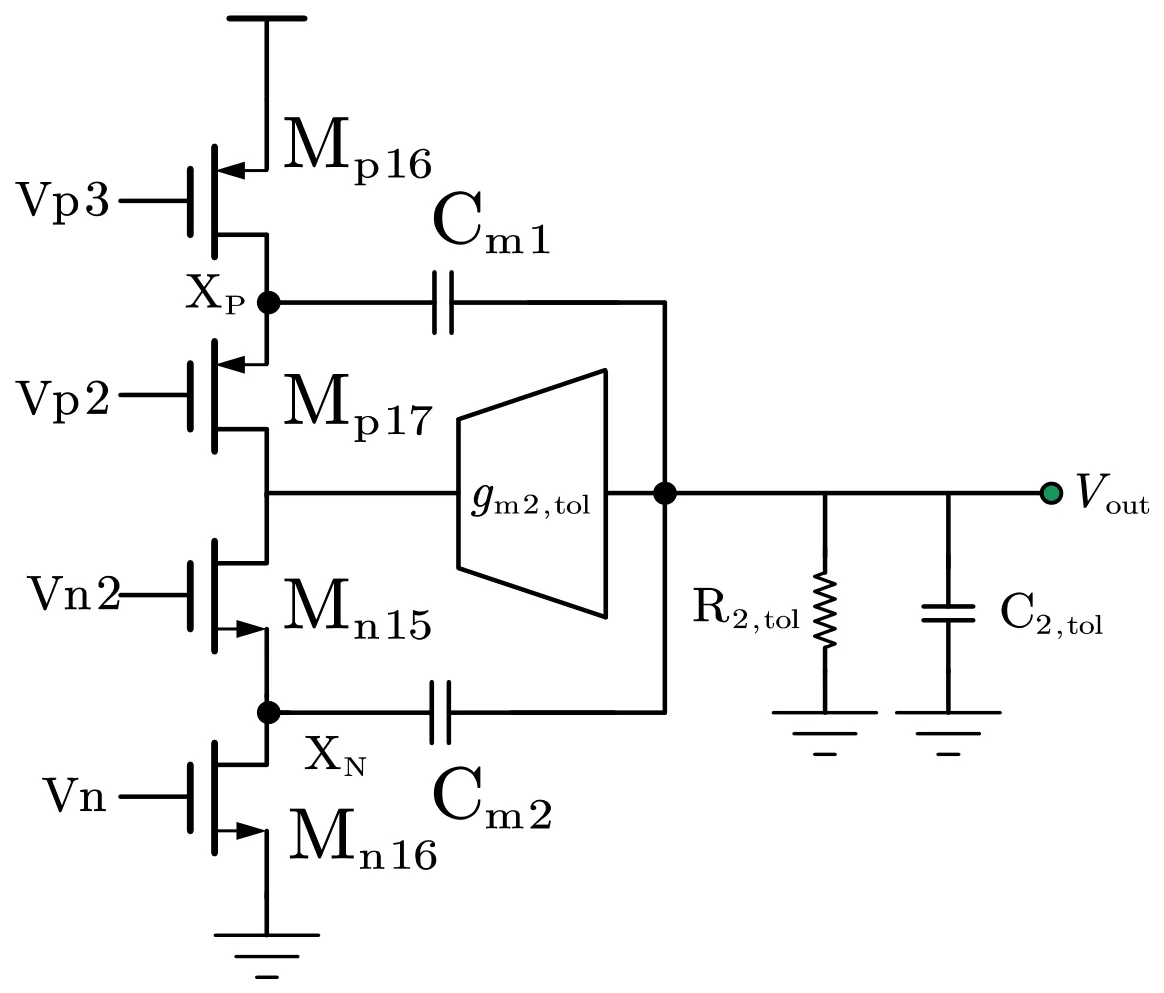
Schematic diagram of cascode compensation.

Conventional Miller compensation realizes dominant-pole compensation through the pole-splitting effect. However, because the compensation capacitor C_C is directly connected between the first-stage high-impedance output node and the second-stage output node, it bypasses the second-stage transconductance path and forms a feedforward path. This feedforward path usually introduces a right-half-plane zero, resulting in additional phase lag, a reduced phase margin, and degraded system stability. To eliminate this right-half-plane zero, the conventional approach typically requires a series zero-nulling resistor. Since this work adopts a cascode structure, cascode compensation is employed to avoid the stability issue caused by the direct feedforward path in conventional Miller compensation, as illustrated in Figure 7. The compensation capacitors are connected from the low-impedance nodes of the PMOS cascode current-source branch and the NMOS cascode current-source branch to the output node, respectively. Because the compensation current is first injected into the low-impedance source node of the cascode device and then affects the output node through the current-buffering action of the cascode branch, this structure can significantly suppress the direct feedforward effect associated with conventional Miller compensation. In addition, the output stage in this work is not a fixed-size structure, but consists of multiple adaptive-output units connected in parallel. As the number of enabled output units m changes, the equivalent transconductance, output resistance, and output parasitic capacitance of the output stage also change, causing the locations of the non-dominant pole and zero to move with the output-stage configuration. Therefore,

(8) $$\begin{array}{cc} g_{m2,tot} & =m\left(g_{mPout,i}+g_{mNout,i}\right)\\ R_{2,tot} & =\frac{r_{oPout,i}//r_{oNout,i}}{m}\\ C_{2,tot} & =m\left(C_{Pout,i}+C_{Nout,i}\right) \end{array}$$

where m denotes the number of currently enabled adaptive-output units; g_mPout,i_ and g_mNout,i_ represent the small-signal transconductances of the PMOS output transistor and NMOS output transistor in a single adaptive unit, respectively; and r_oPout,i_ and r_oNout,i_ represent their small-signal output resistances, respectively. As m increases, both the equivalent transconductance and the equivalent output conductance of the output stage increase, thereby enhancing the output driving capability.

Assuming that the equivalent transconductance of the first-stage operational amplifier is g_m1_, and that its equivalent resistance and capacitance are R_1_ and C_1_, respectively, the first pole of the operational amplifier can be approximately expressed as follows:

(9) $$\omega_{p1}\approx \frac{1}{R_{1}\left[C_{1}+\left(C_{m1}+C_{m2}\right)\left(1+g_{m2,tot}R_{2,tot}\right)\right]}$$

When the second-stage gain satisfies Av2 >> 1, and C_m1_ + C_m2_ >> C_1_, it can be further simplified as follows:

(10) $$\omega_{p1}\approx \frac{1}{R_{1}\left(C_{m1}+C_{m2}\right)g_{m2,tot}R_{2,tot}}$$

The non-dominant pole corresponding to the output stage can be approximately expressed as follows:

(11) $$\omega_{p2}\approx \frac{g_{m2,tot}}{2C_{m1}+C_{2,tot}}$$

## 4. Test Results and Analysis

To verify the feasibility and performance of the proposed adaptive-output operational amplifier architecture, complete circuit simulations were carried out on the Cadence Virtuoso platform using a 0.18 µm CMOS process. The simulation setup is as follows: the supply voltage *V_DD_* is 3.0 V and the common-mode input voltage *V_cm_* is 1.5 V. According to the design targets, the threshold voltages are set to *Ref_H_* = 1.51 V (high threshold), *Ref_L_* = 1.49 V (low threshold), and *Ref_M_* = 1.50 V (mid-supply reference voltage). Two load configurations were used in the simulations. A variable resistive load was first used as a controlled current-demand stimulus to verify the adaptive enabling and disabling behavior of the output-stage array. This resistive-load condition is not intended to represent the physical impedance of the MEMS drive electrode. Instead, it provides a direct way to impose different source/sink current requirements on the operational amplifier output and observe the corresponding threshold-detection and shift-register response. To evaluate the stability under a more representative MEMS drive condition, an equivalent capacitive load was further introduced at the amplifier output, as discussed below.

**Figure 8:**
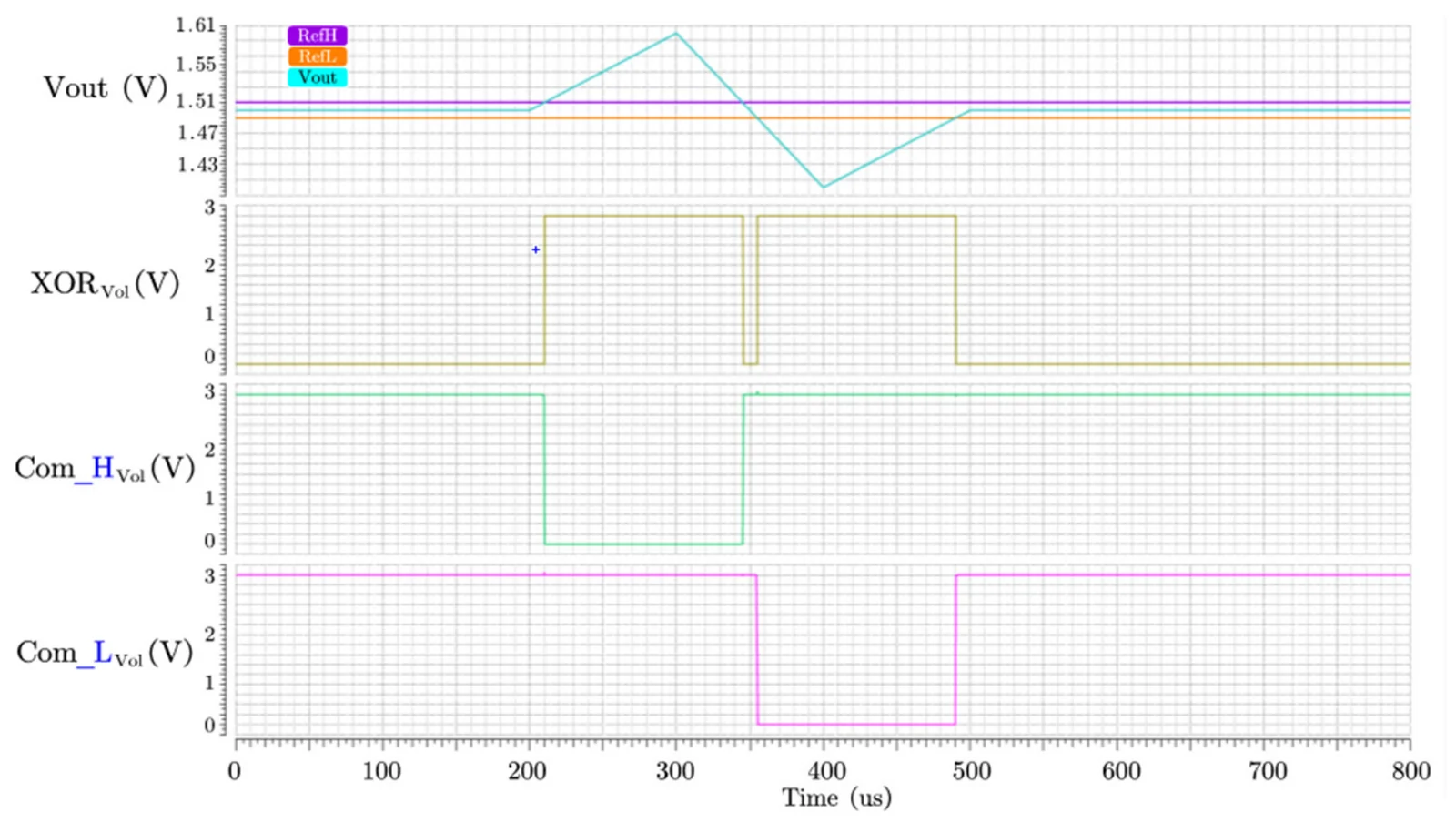
Simulation Results of Voltage Detection Circuit.

The simulation results of the voltage detection circuit are shown in Figure 8. The results indicate that when the output voltage *V_out_* lies between *Ref_L_* and *Ref_H_*, the XOR gate outputs logic “0”. This corresponds to the stable region defined by the system, in which the shift-control clock is masked and the circuit remains idle. When *V_out_* goes beyond this range, the comparators toggle rapidly and the XOR output becomes logic “1”, thereby activating the subsequent control circuitry. All current values reported in this paper refer either to simulated load conditions or measured supply currents, as explicitly indicated.

**Figure 9:**
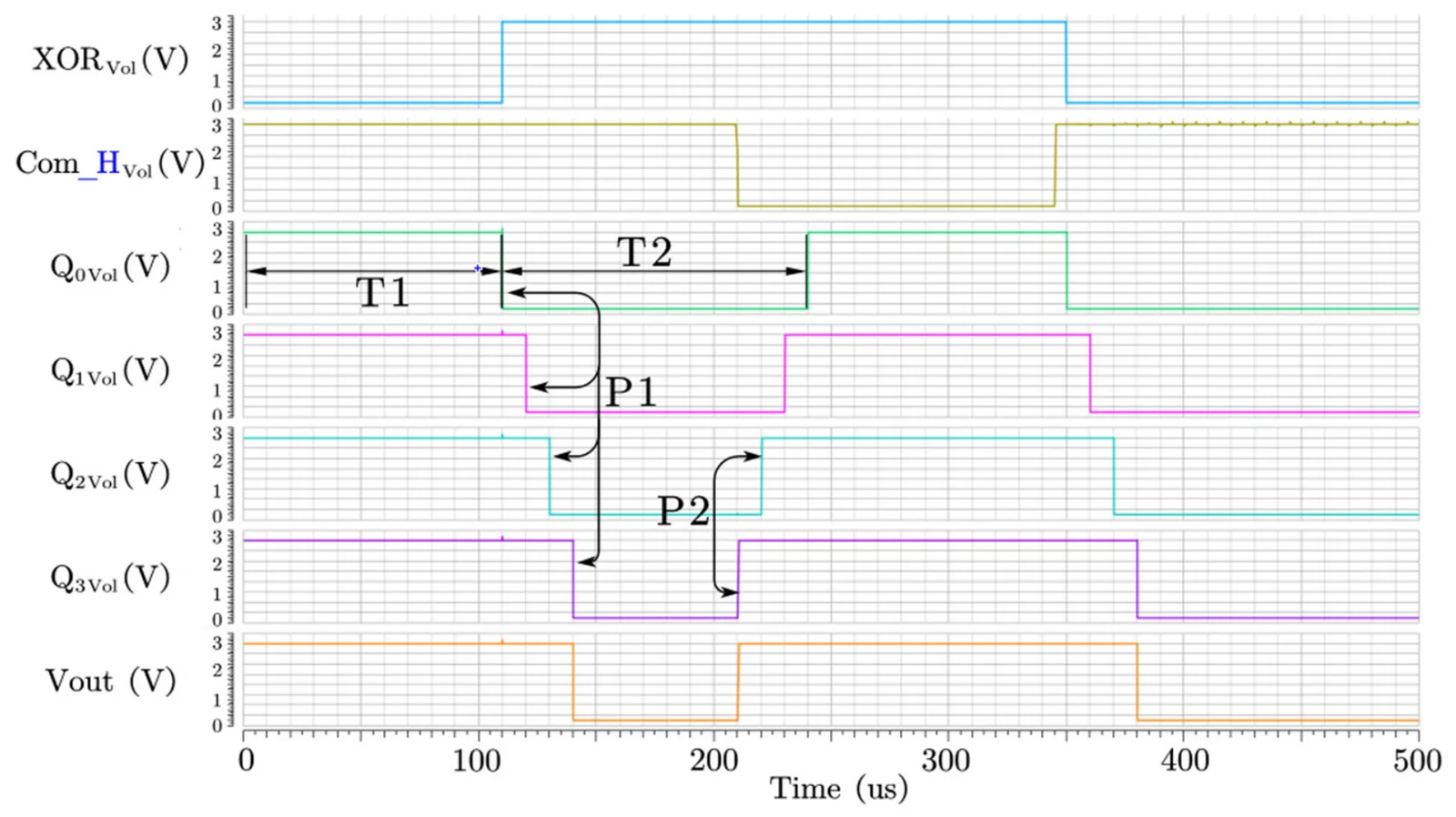
Simulation results of the bidirectional shift register. The blue, yellow, green, magenta, cyan, purple, and orange lines represent $XOR_{Vol}$, $Com\text{\_}H_{Vol}$, $Q_{0Vol}$, $Q_{1Vol}$, $Q_{2Vol}$, $Q_{3Vol}$, and $V_{out}$, respectively.

The bidirectional shift-register simulation waveform is shown in Figure 9. It can be observed that in the initial stage, when the XOR output is 0, the shift register defaults to output “1”; in this case, the register drives the SPDT switch to turn on the static output transistors. When the XOR output becomes 1, the shift register shifts left or right (P1, P2) under the control of the threshold comparators. During *T*_1_, an under-drive condition is detected and the shift register performs a right-shift operation driven by the clock, indicating that the system is progressively enabling PMOS power units to increase pull-current capability. During *T*_2_, an “over-drive” condition after load relaxation (i.e., *V_out_* exceeds *Ref_M_*) is emulated; within the decision window, the shift register performs a left-shift operation to progressively disable higher-order outputs. These results demonstrate that the circuit can proactively withdraw redundant drive when the load becomes lighter. To further validate the effectiveness of the two-stage control strategy under complex operating conditions, detailed transient response waveforms of the system from *T*_3_ to *T*_7_ are extracted. These waveforms capture the complete closed-loop process, from large-current establishment to steady-state locking and then to energy-efficiency optimization.

**Figure 10:**
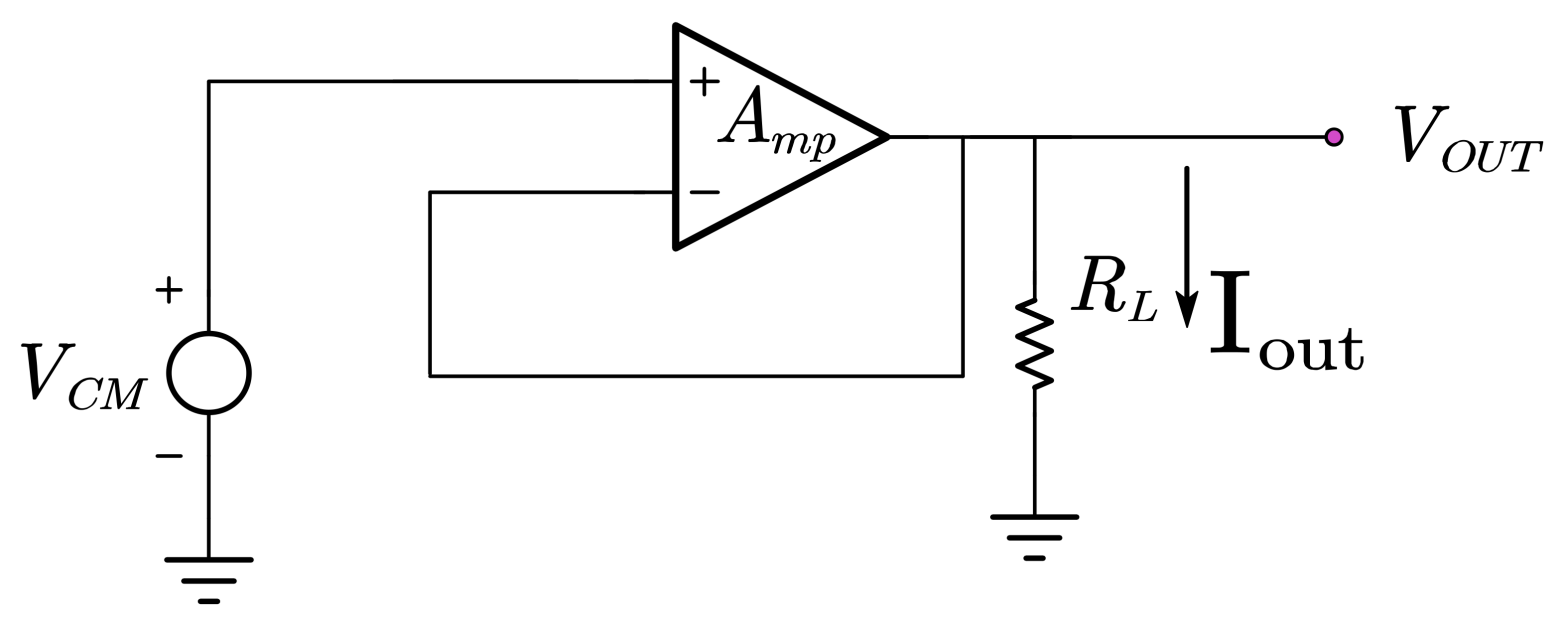
Unity-gain buffer testbench for adaptive-output transient simulation.

Finally, a full-system simulation is performed. The simulation testbench is shown in Figure 10, where the operational amplifier is configured as a unity-gain buffer, forcing the output to *V*_OUT_ ≈ *V_CM_*.

**Figure 11:**
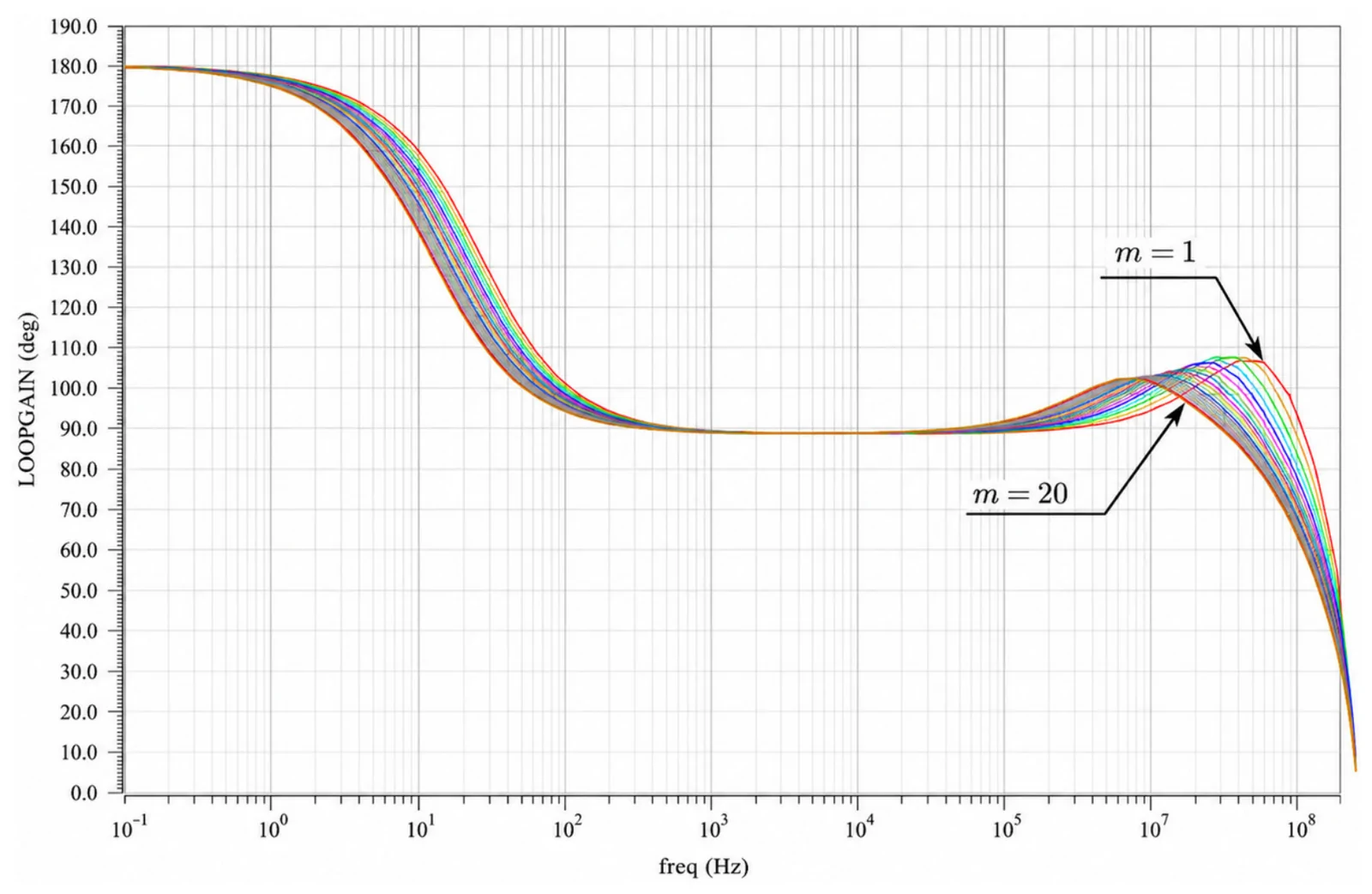
Frequency compensation simulation.

Because both ω_p1_ and ω_p2_ are affected by the output-array configuration and parasitic parameters, the worst-case stability condition cannot be accurately determined using only analytical approximations. Therefore, STB simulations under different adaptive output configurations were further performed in Cadence Virtuoso. During the simulations, the shift register was fixed, and different numbers of enabled output units m were set. The simulation results are shown in the Figure 11.

It can be observed that, as the number of enabled output units m increases, the phase-lead behavior in the phase curve appears at a lower frequency, indicating that the zero location or non-dominant pole location of the system shifts with the output-stage configuration. This phenomenon is mainly related to the gate-drain parasitic capacitance of the adaptive output transistors and the equivalent conductance at the output node. On the one hand, enabling more output transistors increases the equivalent gate-drain feedforward capacitance, which shifts the associated left-half-plane zero to a lower frequency and therefore provides the phase lead earlier. On the other hand, enabling more output transistors also increases the equivalent conductance at the output node, pushing the output-related non-dominant pole to a higher frequency and reducing the phase lag around the unity-gain frequency. These two effects jointly increase the phase margin as m increases.

Although the STB simulation verifies the small-signal stability under fixed output-stage configurations, it does not directly evaluate the nonlinear switching behavior caused by the adaptive enabling and disabling of output cells. Therefore, a transient stability simulation was further performed to examine whether the proposed threshold-based control may introduce repetitive switching or limit-cycle oscillation near the threshold boundaries.

**Figure 12:**
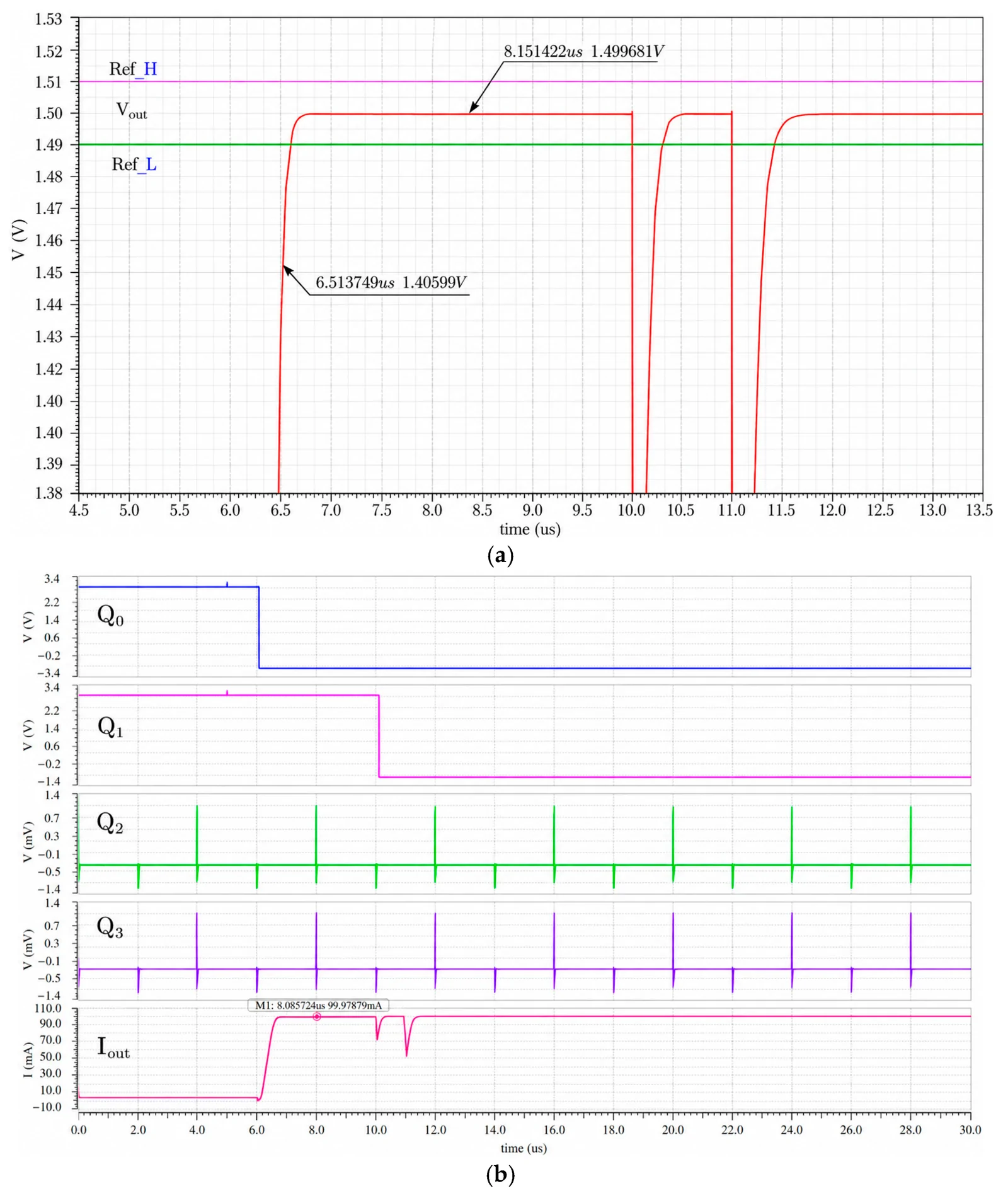
Transistor-level transient simulation of nonlinear adaptive pull-up switching. (**a**) Output voltage V_out_ and the reference thresholds RefH and RefL. (**b**) Voltage-detection output, simulated shift-register states Q0–Q3, and simulated output current I_out_.

The internal enable states of the adaptive-output array are not directly accessible during the MEMS gyroscope measurement. Therefore, the measured gyroscope oscillation is used only to verify that the proposed drive amplifier can support stable closed-loop resonant operation. The adaptive enable/disable behavior is verified separately through a transistor-level transient simulation, where the voltage-detection output, shift-register states, enabled output units, and output current can be observed. To evaluate the transient stability of the adaptive output regulation under nonlinear switching conditions, a pull-up load transient simulation was performed. The operational amplifier was configured as a unity-gain buffer, and the output node was connected to a time-varying load to emulate a sudden increase in source-current demand. As shown in Figure 12a, the output voltage initially drops below RefL, indicating that the available pull-up capability is insufficient. The voltage-detection circuit therefore identifies an under-drive condition and activates the PMOS-side shift-register control.

The corresponding enable states and output current are shown in Figure 12b. During the under-drive interval, the PMOS output units are enabled step by step at the control-clock edges, and Iout increases accordingly. As the pull-up current increases, V_out_ gradually recovers toward the threshold window. Once V_out_ returns to the region between RefL and RefH, the shift register retains the current enable word, and no further PMOS output units are activated. The output current also reaches a stable value corresponding to the applied load condition. These results confirm that the proposed adaptive output regulation completes only finite state transitions during the out-of-range interval. After the output voltage returns to the threshold window, the output-stage configuration is retained rather than repeatedly toggled. Therefore, no persistent current spikes, repetitive enable-state transitions, or sustained limit-cycle oscillation are observed in the nonlinear switching transient.

In an electrostatic MEMS gyroscope, the drive electrode is primarily a capacitive load rather than a resistive load. The electrical load seen by the drive amplifier can be approximated as the MEMS drive-electrode capacitance together with routing, pad, and interface parasitic capacitances, expressed as follows:

(12) $$C_{L}=C_{\mathrm{MEMS}}+C_{\mathrm{par}}$$

The electrostatic drive force is related to the drive voltage by

(13) $$F_{\mathrm{elec}}=\frac{1}{2}\frac{dC(x)}{dx}V_{\mathrm{drive}}^{2}$$

Indicating that the mechanical driving force is nonlinear with respect to the voltage. Therefore, the resistive-load simulation is used only to verify the output-current-regulation mechanism, while the capacitive-load simulation is used to evaluate the stability of the amplifier under a MEMS-equivalent electrode load.

Additional STB simulations were performed by connecting capacitive loads at the amplifier output. The operational amplifier was configured as a unity-gain buffer with VDD = 3 V and VCM = 1.5 V. The load capacitance was swept from 10 pF to 50 pF to emulate the equivalent MEMS drive-electrode capacitance together with the parasitic capacitance introduced by interconnects, pads, and interface nodes. This capacitive-load condition directly evaluates the additional phase shift introduced at the amplifier output.

The simulated STB results are summarized in Table 1. As CL increases from 10 pF to 50 pF, the phase margin decreases from 93.582° to 85.062°, indicating that the capacitive load introduces additional phase lag, as expected. Meanwhile, the gain-margin frequency shifts from 90.041 MHz to 39.995 MHz, which is consistent with the movement of the output-related pole caused by the increased capacitive loading. Nevertheless, over the entire simulated capacitance range, the phase margin remains higher than 85°, and the gain margin remains higher than 12.7 dB. These results confirm that the proposed adaptive-output operational amplifier maintains a sufficient stability margin under the simulated MEMS-equivalent capacitive-load conditions.

To quantitatively evaluate the energy-efficiency improvement provided by the proposed adaptive output regulation, the supply current and average power consumption were compared with those of a fixed-output-stage counterpart. In the fixed-output-stage configuration, all adaptive-output units were kept enabled to provide a constant high-drive capability, while the main amplifier, bias circuit, compensation network, supply voltage, and load conditions were kept unchanged. Therefore, this comparison directly reflects the power reduction achieved by dynamically disabling redundant output units.

The supply current was extracted from the 3 V voltage source in the transient simulation. The output load current was used only to define the load condition, whereas the power consumption was calculated from the supply current. The average power consumption was calculated as

(14) $$P_{\mathrm{avg}}=V_{DD}\cdot \frac{1}{T}\int_{0}^{T}I_{DD}(t)dt$$

The light-load condition corresponds to I_load_ = 1 mA, for which the intrinsic Class-AB output stage can maintain Vout within the (Ref_L)–(Ref_H) window without enabling additional adaptive output units. The regulated heavy-load condition corresponds to I_load_ = 3 mA, for which additional adaptive output units are enabled and then retained after Vout returns to the threshold window. To avoid possible misunderstanding, it should be emphasized that the previously reported 400 mA current does not represent the actual continuous output current delivered by the proposed operational amplifier. Instead, it originated from an intermediate design description and could be misinterpreted without sufficient explanation. Since this value does not correspond to the practical operating condition of the fabricated circuit, the related statement has been removed in the revised manuscript. The adaptive-output mechanism is evaluated using representative load-current conditions that emulate different output driving requirements. Therefore, the proposed architecture should be understood as a dynamically configurable output-stage design for improving power efficiency rather than a high-current output amplifier intended to continuously source hundreds of milliamperes.

The previous description of the 400 mA current has been corrected to avoid ambiguity. Throughout the revised manuscript, the adaptive-output capability is evaluated under representative operating conditions that are consistent with the intended application of electrostatic MEMS gyroscope driving circuits.

The power-comparison results are summarized in Table 2. Under the light-load condition, the proposed adaptive-output design reduces the average power consumption from 4.007 mW to 3.212 mW, corresponding to a (19.84%) reduction compared with the fixed-output-stage counterpart. Under the regulated heavy-load condition, the average power consumption is reduced from 10.92 mW to 10.04 mW, corresponding to an (8.06%) reduction. These results confirm that the proposed adaptive output regulation reduces unnecessary static power consumption under light-load operation while preserving sufficient drive capability when the load demand increases.

The statistical results of the key performance metrics are summarized in Table 3. In terms of process-corner verification, PVT simulations were performed, covering three process corners (TT/SS/FF), three supply voltages (2.7/3.0/3.3 V), and three temperatures (−40/27/125 °C), resulting in a total of 27 conditions. The results show that the gain varies from 104.046 to 115.09 dB, the GBW ranges from 11.897 to 22.7 MHz, the phase margin varies from 79.91° to 86.35°, and the slew rate ranges from 11.4 to 24.8 V/μs. The worst-case conditions mainly occur at a low supply voltage, a high temperature, and the SS process corner. Nevertheless, the circuit remains stable under all tested conditions, with the phase margin always greater than 79°, indicating that the design exhibits good robustness against process, voltage, and temperature variations. The detailed parameters are listed in Table 4.

**Figure 13:**
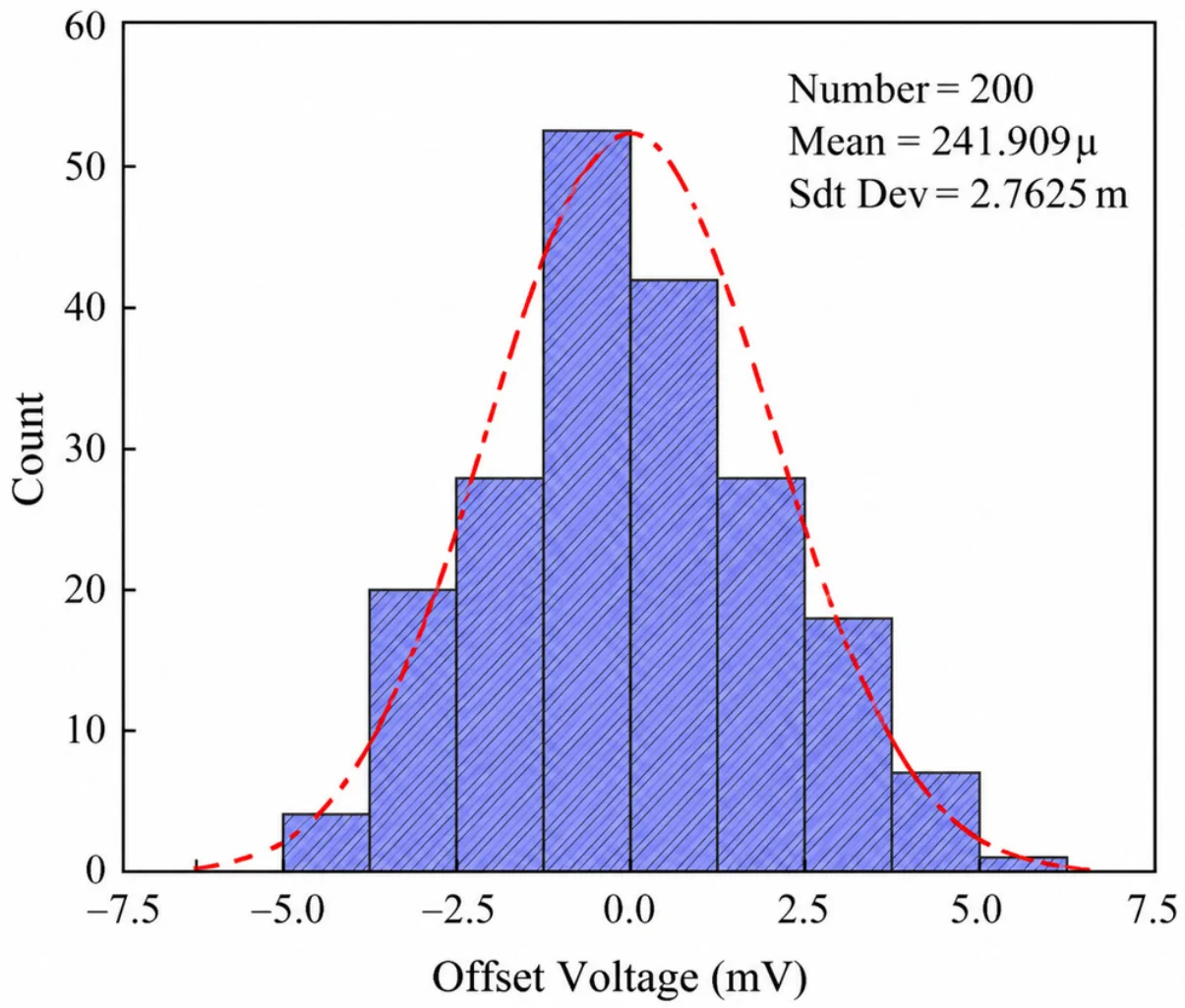
Monte Carlo Simulation of the Operational Amplifier Offset Voltage.

The simulated input-referred offset has a mean value of 241.9 μV and a standard deviation of 2.76 mV. Since the nominal threshold window is RefM ± 10 mV, the 3σ offset level is approximately 8.28 mV, which remains below the nominal half-window. Therefore, under the simulated mismatch condition, the offset mainly shifts the effective switching boundary rather than eliminating the detection window. However, when V_out_ is very close to RefH or RefL, comparator offset and threshold variation may cause early or delayed switching by one or several control-clock cycles. The retained shift-register state helps prevent continuous toggling after V_out_ returns to the RefL–RefH window. This result also indicates that further reduction of the threshold window would require additional offset calibration, hysteresis, or a larger design margin. In addition, Monte Carlo simulations were included to evaluate the impact of device mismatch on the offset voltage, and the statistical results along with the distribution plot have been added to the revised manuscript. Two hundred Monte Carlo runs were performed to assess the input offset voltage of the operational amplifier under process variations and local mismatch conditions as shown in Figure 13. The simulation results indicate that the mean offset voltage is approximately 241.9 μV, with a standard deviation of 2.76 mV. This analysis further illustrates the bias sensitivity and statistical behavior of the circuit under random mismatch conditions. Future work will extend the statistical analysis to the complete adaptive decision path, including comparator offset, reference mismatch, hysteresis design, and false-switching probability.

It should be noted that the proposed adaptive-output mechanism is an internal circuit function implemented by the digital control logic and segmented output stage. Since no dedicated observation nodes were reserved in the fabricated prototype, the internal adaptive switching states cannot be directly measured experimentally. Therefore, transistor-level simulations are employed to verify the adaptive operation, whereas the fabricated chip measurements demonstrate the practical applicability of the proposed amplifier in the electrostatic MEMS gyroscope drive loop.

**Figure 14:**
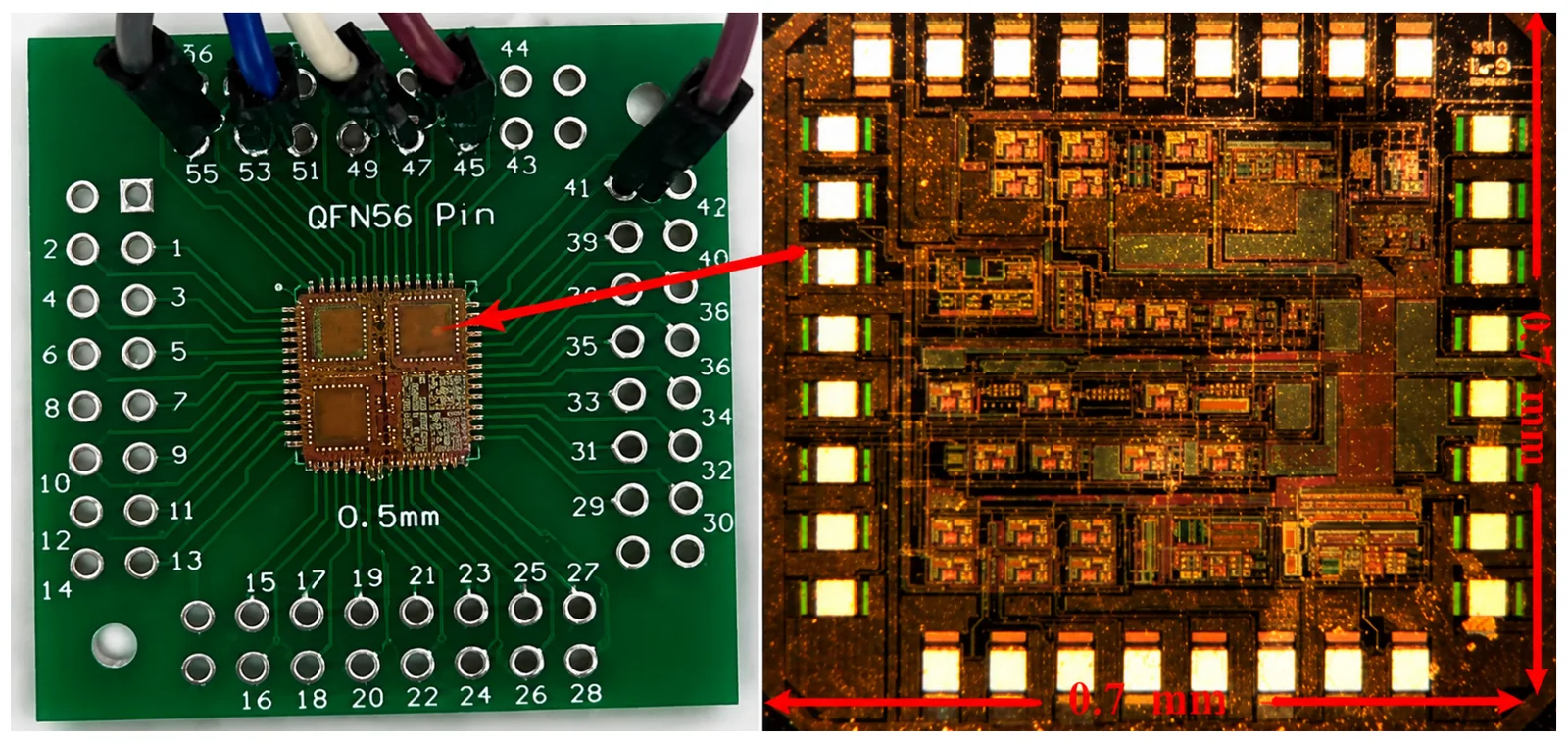
The photograph of the PCB and ASIC chip.

To verify the functionality of the proposed circuit, the adaptive-output operational amplifier for the MEMS gyroscope was fabricated using the SMIC standard 0.18 μm CMOS process. The ASIC chip was mounted on a custom-designed PCB, as shown in Figure 14. The chip area is approximately 0.7 mm × 0.7 mm. Wire bonding was performed using a wedge bonder to connect the 33 pad points on the ASIC chip to the corresponding pads on the PCB with aluminum–silicon bonding wires. The assembled system was then used for experimental testing.

**Figure 15:**
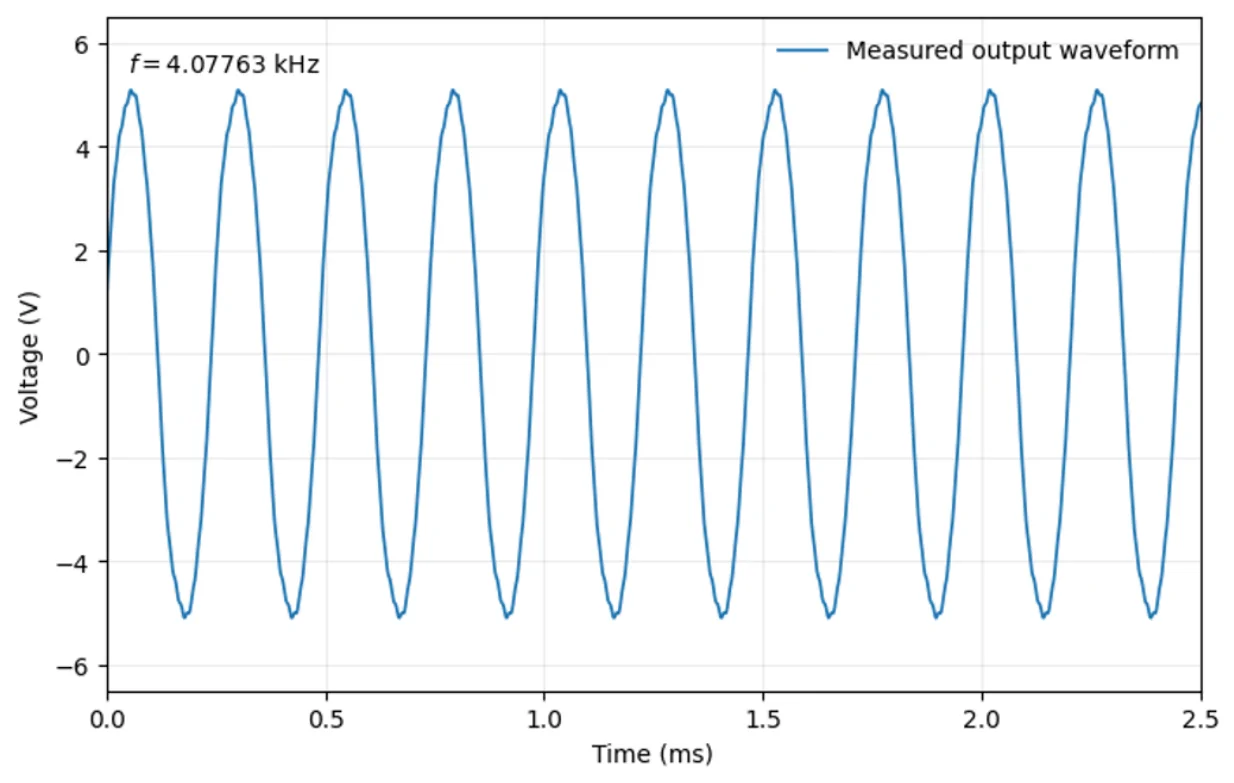
Measured time-domain waveform of the proof-mass velocity signal under electrostatic force-modulated closed-loop self-excited driving.

To evaluate the electrostatic force-modulated closed-loop self-excited driving performance of the proposed MEMS gyroscope interface ASIC, experimental measurements were carried out using a dynamic signal analyzer (HP 35670A, Hewlett-Packard, Palo Alto, CA, USA) and an oscilloscope (TDS2012B, Tektronix, Beaverton, OR, USA). In the proposed driving scheme, a high-frequency square-wave carrier generated by the ASIC was employed to modulate the proof-mass velocity signal, and the modulated driving voltage was subsequently applied to the gyroscope driving comb electrodes to realize electrostatic force excitation. The measured proof-mass velocity signal waveform based on the electrostatic force-modulated closed-loop self-excited driving principle is shown in Figure 15. It can be observed that the gyroscope successfully achieves stable self-excited oscillation under atmospheric pressure conditions. The measured waveform exhibits a highly periodic and continuous resonant signal with no obvious amplitude collapse, waveform distortion, or oscillation interruption, indicating that the proposed interface ASIC can effectively sustain resonant motion through closed-loop electrostatic force compensation. Moreover, the measured resonant frequency is approximately 4.077 kHz, which agrees well with the designed operating condition of the gyroscope resonator. The stable sinusoidal-like waveform and consistent oscillation period further demonstrate that the proposed closed-loop driving circuit is capable of maintaining steady resonant operation and suppressing the resonance instability caused by environmental disturbances or energy dissipation. These results verify the feasibility and effectiveness of the proposed electrostatic force-modulated self-excited drive architecture for MEMS gyroscope applications.

**Figure 16:**
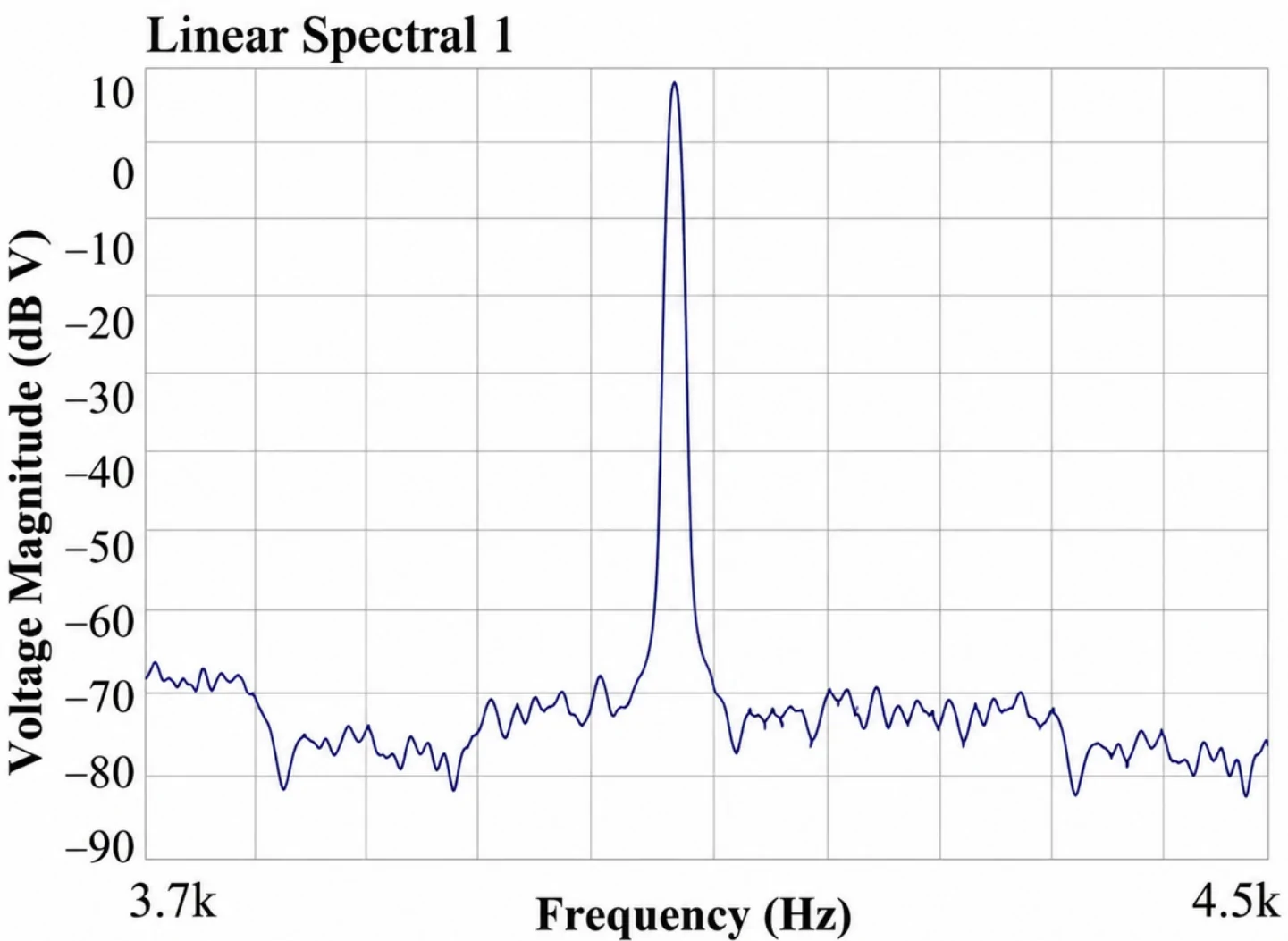
Measured frequency spectrum of the proof-mass velocity signal under electrostatic force-modulated closed-loop self-excited driving.

The frequency-domain response of the proof-mass velocity signal under electrostatic force-modulated closed-loop self-excited driving was further characterized using a dynamic signal analyzer (HP35670A), and the measured spectrum is shown in Figure 16. A distinct and sharp resonance peak can be observed at approximately 4.077 kHz, indicating that the proposed interface ASIC successfully excites and maintains stable resonant oscillation of the MEMS gyroscope under atmospheric pressure conditions. The measured spectrum exhibits strong spectral concentration around the resonant frequency with a relatively low noise floor and no noticeable parasitic oscillation components, demonstrating the effective suppression of resonance disturbances and stable closed-loop operation. To quantitatively evaluate the oscillation stability, the displacement amplitude stability of the proof mass was measured using the voltage mode of an Agilent 34410A digital multimeter. The measured voltage fluctuation was below 0.2 mV relative to an AC driving amplitude of 5.7 V, corresponding to an amplitude stability of approximately 0.005%. In addition, the frequency stability of the velocity signal was evaluated using the frequency measurement mode of the digital multimeter (Agilent 34410A, Agilent Technologies, Santa Clara, CA, USA) Experimental results show that the resonant frequency remained centered at 4.077 kHz with a fluctuation lower than 0.1 Hz over a 15 min observation period, corresponding to a relative frequency stability of approximately 25 ppm. These results demonstrate that the proposed electrostatic force-modulated closed-loop self-excited driving architecture achieves stable amplitude and frequency control, enabling robust and reliable operation of the MEMS gyroscope under atmospheric conditions.

We have added a comparison table with existing works, as shown in Table 5. The results demonstrate that the proposed design achieves high gain, a large phase margin, and good CMRR/PSRR performance in a 180 nm process, while also providing a certain level of load-driving capability. The present comparison focuses on circuit-level verification rather than complete system-level energy characterization.

## 5. Conclusions

The proposed adaptive-output operational amplifier was implemented and evaluated as a circuit block for the electrostatic closed-loop drive path of an MEMS gyroscope. Circuit-level simulations verify the adaptive output regulation, capacitive-load stability, nonlinear switching behavior, and supply-power reduction relative to a fixed-output-stage counterpart. The MEMS gyroscope measurement confirms the stable closed-loop resonant operation supported by the proposed drive amplifier, while the internal adaptive enable/disable behavior is verified through a transistor-level simulation. By employing a three-threshold decision mechanism with RefH = 1.51 V, RefL = 1.49 V, and RefM = 1.50 V, together with a two-stage timing-regulation strategy constrained by the detection period Tdet and the control period Tctrl, the proposed design achieves both a rapid transient response and stable steady-state operation under dynamically varying electrostatic drive demands. During start-up, resonance establishment, and abrupt disturbance conditions, the bidirectional shift register activates additional output power units step by step within a finite number of control cycles, thereby enhancing electrostatic driving capability and rapidly restoring closed-loop oscillation stability. Once stable resonance is established, redundant output branches are progressively disabled based on the mid-supply reference voltage RefM, effectively suppressing unnecessary static power dissipation while maintaining stable oscillation. Experimental results obtained using the MEMS gyroscope interface ASIC demonstrate that the proposed electrostatic-force-modulated closed-loop self-excited driving scheme successfully achieves stable resonant operation under atmospheric pressure conditions. The measured resonant frequency is centered at 4.077 kHz, with a fluctuation lower than 0.1 Hz over a 15 min observation period, corresponding to a relative frequency stability of approximately 25 ppm. Moreover, the proof-mass displacement amplitude fluctuation remains below 0.2 mV relative to an AC driving amplitude of 5.7 V, corresponding to an amplitude stability of approximately 0.005%. Combined with the hard shut-off isolation of idle power transistors enabled by the improved SPDT switch, the subthreshold leakage current is effectively suppressed, further improving system energy efficiency. These results verify the effectiveness of the proposed design in jointly optimizing electrostatic driving capability, oscillation stability, and power efficiency, thereby providing a practical and reliable solution for high-performance MEMS gyroscope electrostatic-force-modulated closed-loop self-excited driving systems.

One limitation of the present work is that the adaptive output-state transitions were verified through transistor-level simulations rather than direct chip measurements because the fabricated prototype did not include dedicated observation circuitry. Future work will focus on redesigning the prototype to experimentally characterize the internal adaptive regulation mechanism and complete system-level power consumption and include a redesigned prototype incorporating dedicated observation nodes for experimentally monitoring the adaptive output-unit states, shift-register transitions, and dynamic power regulation during practical MEMS operation.

## Figures and Tables

**Table 1:** Stability simulation results under capacitive loads.

CL	PM (deg)	PM Frequency	GM (dB)	GM Frequency
10 pF	93.582	8.3889 MHz	14.182	90.041 MHz
20 pF	91.595	8.2707 MHz	13.508	64.078 MHz
30 pF	89.495	8.4847 MHz	13.226	52.078 MHz
40 pF	87.294	8.5966 MHz	12.742	44.423 MHz
50 pF	85.062	8.6341 MHz	12.966	39.995 MHz

**Table 2:** Power-consumption comparison between the adaptive and fixed-output-stage amplifiers.

Condition	Load Current	I_DD,avg_	P_avg_	I_DD,avg,fixed_	P_avg,fixed_
Light load	Iout = 1 mA	1.071 mA	3.212 mW	1.336 mA	4.007 mW
Regulated heavy load	Iout = 3 mA	3.347 mA	10.04 mW	3.640 mA	10.92 mW

**Table 3:** Operational Amplifier Performance Parameters.

Performance Parameters	Simulation Results
GBW	17.14 MHz
Phase Margin	85.73 Deg
Gain	109 dB
SR	14.5 V/μs
Output range	0–2.42 V
CMRR	93 dB
PSRR	115 dB

**Table 4:** Performance of the Operational Amplifier under Different PVT Conditions.

Parameter	Min	Max	Worst Corner
DC Gain (dB)	104.046	115.09	ff, 2.7, 125 °C
GBW (MHz)	11.897	22.7	ss, 2.7, 125
Phase Margin (deg)	79.91	86.35	ss, 2.7, 125
Slew Rate (V/μs)	11.4	24.8	ss, 2.7, 27

**Table 5:** Comparison with Reported Works.

Parameter	This Work	[28]	[29]	[30]
Supply(V)	3	1.8	1.3–2.5	0.4
CMRR(dB)	93	81	/	76.28
PSRR(dB)	115	94	/	/
DC gain(dB)	109	80	85	70.26
Output range(V)	0.2–2.8	/	0–0.55	/
Phase Margin	85	63	57	61
Gain Bandwidth(MHz)	17.14	0.214	/	1.21
Slew Rate(V/μs)	14.5	2.3	/	17.07
Process(nm)	180	180	65	180

## Data Availability

The original contributions presented in this study are included in the article. Further inquiries can be directed to the corresponding author.
